# Autonomous Device for Application in Late-Phase Hemorrhagic Shock Prevention

**DOI:** 10.1371/journal.pone.0089903

**Published:** 2014-02-24

**Authors:** Vlad Oncescu, Seoho Lee, Abdurrahman Gumus, Kolbeinn Karlsson, David Erickson

**Affiliations:** 1 Sibley School of Mechanical and Aerospace Engineering, Cornell University, Ithaca, New York, United States of America; 2 Electrical and Computer Engineering, Cornell University, Ithaca, New York, United States of America; University of Hong Kong, Hong Kong

## Abstract

Hemorrhagic shock (HS) is the leading cause of death for people with traumatic injuries. The onset of HS is correlated with marked changes in the plasma vasopressin levels and some studies indicate that administrating vasopressin in the bloodstream can help stabilize the situation. This situation calls naturally for the use of implantable devices for both the monitoring and treatment of HS. In this work, we present a self-powered hemorrhagic-shock autonomous integrated device (hemoAID) that continuously monitors vasopressin levels and releases vasopressin automatically when levels drop below a certain threshold. We demonstrate that the device can operate at physiological concentrations of vasopressin, in sheep serum, thus paving the way towards the development of an autonomous implantable device for HS prevention.

## Introduction

Over the past decade, implantable autonomous microsystems have been developed to counter life-threatening medical conditions and to improve patient care by replacing cumbersome treatment procedures [1]–[3]. As such, a growing number of patients now depend on implantable cardioverter defibrillators [4], gastric stimulators [5], cardiac pacemakers [6] and artificial organs [7]–[9] for survival. In such cases, the severity and urgency of the medical condition override the high cost and risk of the invasive surgery that is required. On the other hand, some situations call naturally for implantable medical devices to continuously monitor physiological changes in patients or to deliver treatment through pre-determined and repeated drug delivery. For instance, continuous glucose monitoring devices have found their market by increasing the efficacy of diabetes treatment and by helping patients monitor real-time variations in their blood glucose levels due to insulin and food intake, exercise, and other factors. Recently, Farra *et al.* [10] demonstrated an implantable drug delivery microchip that operates for up to 3 weeks and eliminates the need for daily physician-assisted injections for patients with osteoporosis. While these developments have been successful at independently performing sensing [11], [12] and drug delivery [13], developing implantable medical devices capable of achieving both of those functions simultaneously would be suitable for a number of applications [14]. One such case is the detection and treatment of traumatic injuries such as hemorrhagic shock (HS).

Studies have correlated the onset of HS with marked changes in the plasma vasopressin levels [15], [16]. Morales *et al.* [15] have conducted a set of canine experiments where vasopressin levels showed a marked increase at the onset of hemorrhage (to as much as 319 pg/mL) followed by a fall to well below the normal physiological level (29 pg/mL) as hemorrhage progressed. In addition, evidence suggests that replenishment of vasopressin at this stage can reverse hypotension in patients [17], [18]. Krismer *et al.* [19] have reported case studies where dosages of 100–160 IU vasopressin led to hemodynamic stability with sinus rhythm in HS patients within 2 min and patient survival without additional treatment of up to 90 min. Therefore, vasopressin can serve as both a biomarker indicative of a critical injury state and a therapeutic agent for treating it. Developing an implantable autonomous device that can provide initial medical treatment when physician care is not readily available is highly suitable in this case.

In this paper, we present a self-powered hemorrhagic-shock prevention autonomous integrated device (hemoAID) that continuously monitors vasopressin levels in solution and releases vasopressin automatically when vasopressin levels drop below a certain threshold. We demonstrate that the device works at physiological concentrations of vasopressin in sheep serum. This represents the first steps towards the development of an implantable, self-powered microfluidic device for late-phase HS prevention. In the next sections, we introduce the hemoAID and discuss the fabrication and integration of its major components. We then demonstrate the operation of the device during rapid changes in vasopressin concentration and proceed by discussing the characteristics of the sensing, drug delivery and power units. Finally in the “Conclusion and Future Prospective” section, we discuss some of the issues that need to be addressed in order to move toward a fully integrated autonomous implantable hemoAID.

## Materials and Methods

### System Design and Assembly

**Figure 1:**
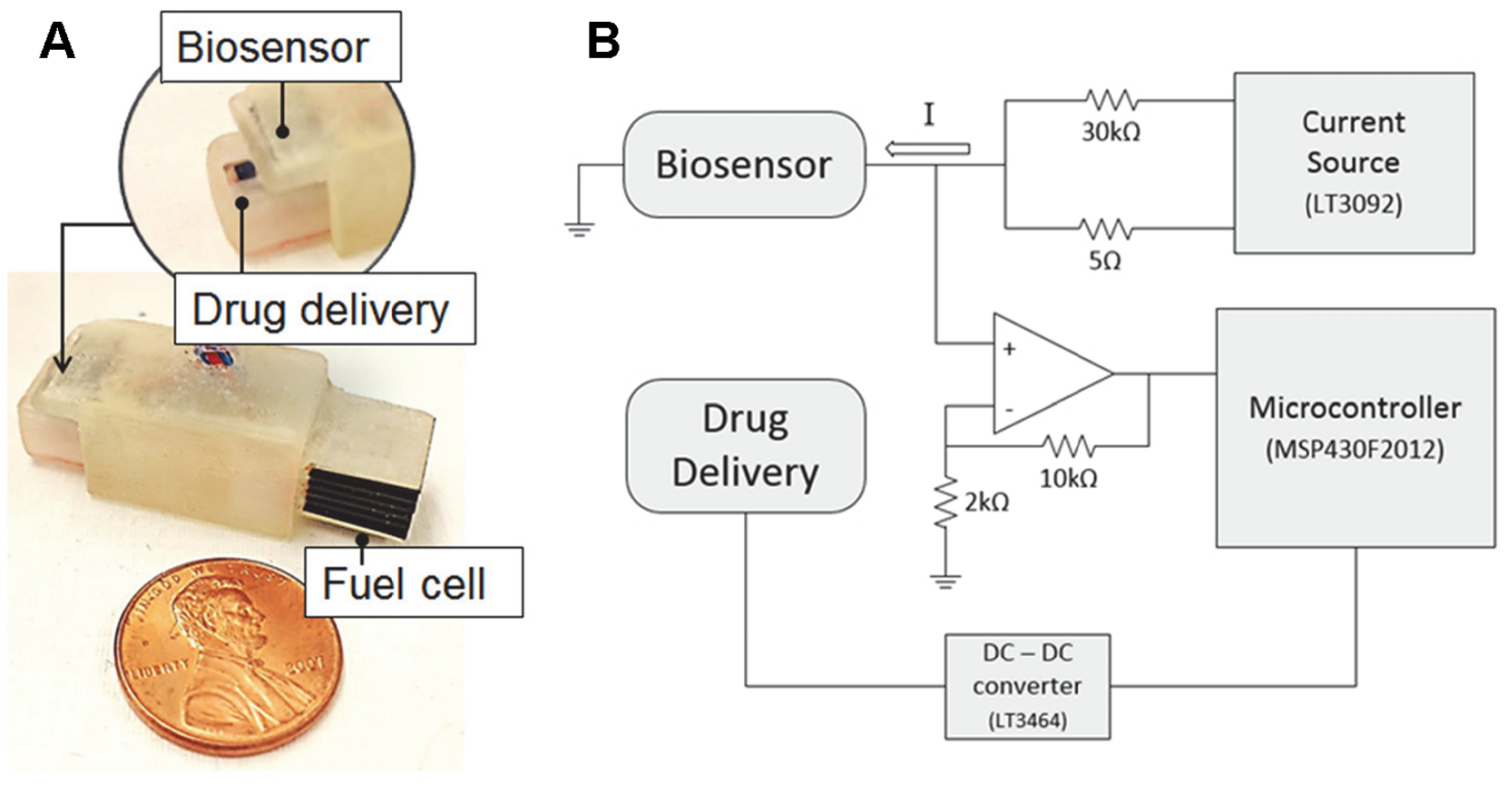
hemoAID device and schematic of electrical connections. A) Integrated device consisting of a biosensor, drug delivery and fuel cell unit B) schematic of the electrical connections between the different components of the hemoAID.

The hemoAID device shown in Fig. 1a consists of a nanowire biosensor for detecting changes in vasopressin levels, an on-board electrochemically driven drug delivery system for administration of vasopressin, and a non-enzymatic glucose fuel cell for powering the system. Those three main functional components are integrated inside a 3 cm^3^ package. In this prototype version, the electronic control system is external to the system and uses the circuit shown in Fig. 1b. External housing of the circuitry was done to facilitate prototype development and could be integrated into the system in the future.

The non-enzymatic glucose fuel cell is used to continuously charge a small rechargeable battery (Thinergy MEC225) that is then used to power a low power microcontroller (MSP430F2012, Texas Instruments, TX, USA). This set-up allows the device to operate steadily even with significant fluctuations in the power output of the fuel cell unit. During device operation, the sensor is driven by an adjustable 2-terminal current source (LT3092, Linear Technology, Milpitas, CA, USA) at 60 mA. The resistance of the sensor changes with the amount of vasopressin in solution, which results in detectable voltage change over the biosensor. In order to improve sensitivity, the voltage is amplified using a non-inverting amplifier (Maxim Integrated, CA) before being detected using a low power microcontroller’s analog to digital converter (ADC). If the voltage is lower than the pre-determined threshold, the microcontroller enables the corresponding output pin to activate the drug delivery system. In order to increase the discharge rate of vasopressin in the drug delivery device, the base voltage (3 V) is converted to 35 V with a micropower step-up DC/DC converter (LT3464, Linear Technology, Milpitas, CA, USA).

### Biosensor Fabrication

The biosensor consists of gold electrodes and a carbon nanotube (CNT) based detection region on which aptamer molecules are immobilized [20]. The detection region of the sensor was patterned on a 4-inch silicon wafer using general photolithography and surface-treated with (3-aminopropyl)trimethoxysilane (APTMS) using Molecular Vapor Deposition Tool (MVD 100). The aminosilanes from the surface treatment were covalently linked with COOH-functionalized multiwalled CNT (Cheap Tubes Inc.) to serve as carriers of aptamer receptors. The scanning electron microscope (SEM) images of immobilized CNT have been reported in our previous work [20]. Electrodes to the detection zone were lithographically patterned, followed by 5 nm chromium/60 nm gold deposition using a CHA Mark 50 e-Beam Evaporator. The wafer was diced into 0.8 cm×0.5 cm pieces which represents significant size reduction from the previous design that measures 5 cm×5 cm.

To immobilize the aptamer molecules on the CNT surface, an activation layer was first created by pipetting a mixture of 0.4 M 1-ethyl-3-(3-dimethylaminopropyl) carbodiimide (Geno Technology) and 0.1 M N-hydroxysuccinimide (MP Biomedicals LLC) in 1∶1 volume ratio. The droplet was maintained for 3 h at room temperature and cleaned with DI H_2_O wash and N_2_ dry. Subsequently, 0.1 µM aptamer solution was pipetted on the surface-treated CNT and maintained for 24 h at room temperature. The premature drying of the droplets for surface modifications was prevented by placing the sensors in a humidified chamber.

### Drug Delivery Device Fabrication

The drug delivery consists of three subcomponents: a top layer with a patterned gold electrode and suspended capping membrane, a bottom layer with a patterned gold counter-electrode and custom packaging to hold the two layers and define a micro-reservoir for vasopressin storage. Fabrication procedures for the top silicon structure are described in details in a previous publication [13]. Briefly, silicon nitride was deposited on both sides of a silicon wafer using low pressure chemical vapor deposition (LPCVD) followed by gold deposition on the top and patterning for reservoir location on the bottom. The wafer was later immersed in potassium hydroxide (KOH) overnight and reactive ion etching was used to remove the silicon nitride in the patterned reservoir location, leaving a 100 µm×100 µm gold capping membrane. Modifications in our new architecture regard the other two components of the device and serve to facilitate subcomponent fabrication and assembly. The replacement of a gold deposited Pyrex layer with a single gold electrode eliminates the standard lift-off processing before the gold deposition. Furthermore, the custom packaging is used in place of PDMS in order to facilitate assembly protocol and reduce leakage of vasopressin through the PDMS. The assembled components were sealed with epoxy adhesive.

### Power Unit Integration

A non-enzymatic glucose fuel cell unit, connected to a rechargeable battery, is used to power the microcontroller and other electrical components of the hemoAID. Lithium batteries were not used here since their short life-time makes them unsuitable for long term autonomous implantable devices with power requirements superior to that of pacemakers [21]. Non-enzymatic glucose fuel cells are promising power sources in such devices due to good long-term stability and adequate power density. Here, we are using the stacked single layer configuration that we have presented previously [22] to demonstrate integration within the hemoAID. The 1 cm^2^ single layer fuel cells are patterned directly on 500 µm thick fused silica substrates. A Raney-type Pt/Ni alloy was used as anode for glucose oxidation and a Pt/Al alloy as cathode for oxygen reduction because of increased selectivity as discussed elsewhere [23], [24]. The fuel cell unit is composed of 12 single layer fuel cells that are connected externally in parallel to form a fuel cell stack and integrated inside of custom holder with a volume of approximately 1 cm^3^.

## Results and Discussion

### Integrated Device Operation in Flow Network

**Figure 2:**
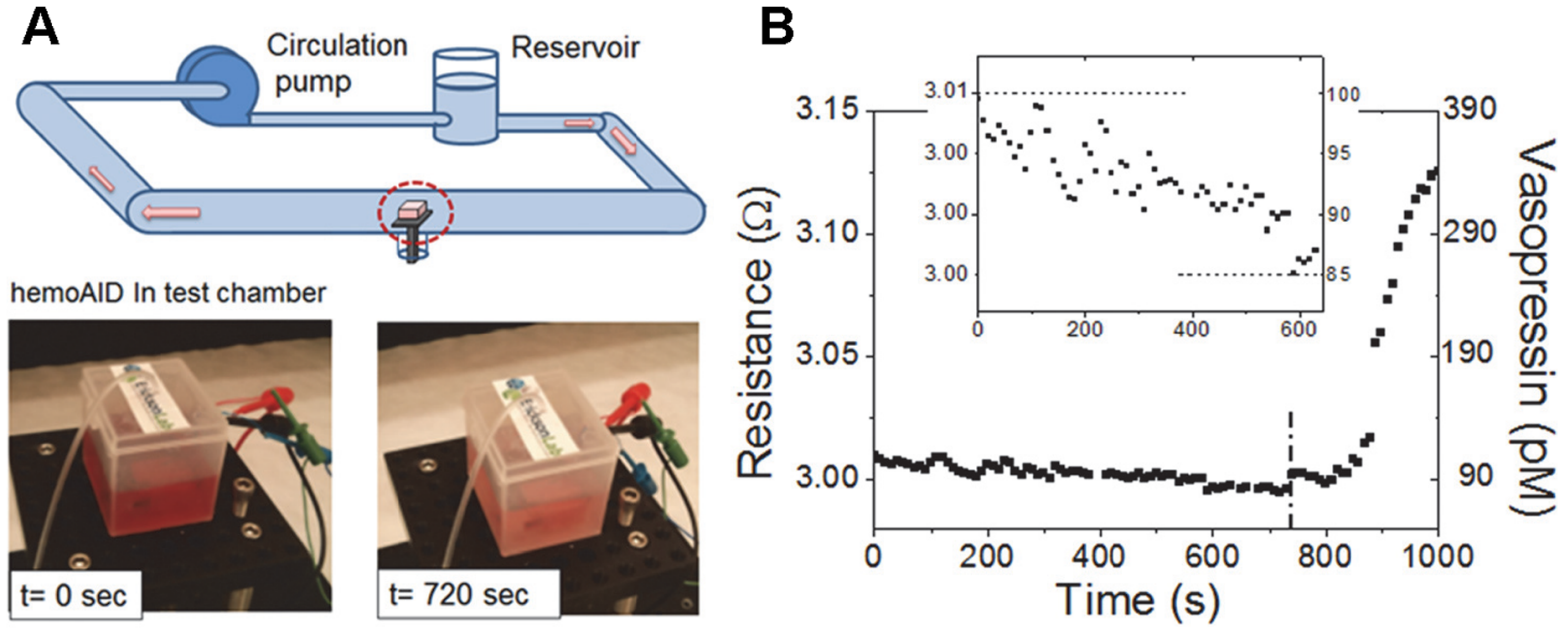
Setup and experiment demonstrating application of hemoAID to late-phase HS prevention. A) Setup for integrated device testing simulating late-phase HS B) resistance change, and associated vasopressin concentration change, detected by the biosensor during integrated experiment simulating late phase HS changes in vasopressin concentration. For the first 720 s the vasopressin concentration is gradually lowered until the drug delivery is autonomously activated to counteract the drop in vasopressin (at 15% drop). The line at 720 s shows the point at which drug delivery is activated. The inset shows the vasopressin drop before the drug delivery is activated (simulating the onset of late-phase HS).

In order to demonstrate its operation, the hemoAID device was placed inside a closed chamber within a fluid network designed to simulate the physiological vasopressin states of late-phase HS patients. According to a clinical study by Jochberger *et al.* [25] vasopressin concentration for patients with hemorrhagic shock rises from the normal 6±3 pM range to 52±30 pM as the body tries to reverse hypotension by releasing vasopressin. Following this initial increase, the level of vasopressin starts decreasing, which indicates the onset of late phase hemorrhagic shock. At that point, administering a high dose of vasopressin can temporarily stabilize the situation. Here, we demonstrate the operation of the hemoAID at those physiologically relevant vasopressin concentrations. The test chamber housing the integrated device (Fig. 2a) is initially filled with 100 pM vasopressin solution, which corresponds to the high initial value (52±30 pM) of blood vasopressin for patients prior to late-phase HS onset. The vasopressin concentration is then progressively lowered by introducing 10 pM solution into the chamber using a VWR Mini-Pump at a flow-rate of 0.115 ml/s. The decrease in vasopressin concentration, indicating late-phase HS, causes a decrease in resistance resulting in a voltage output drop across the nanowire sensor at constant current. This resistance drop across the sensor can be correlated to the vasopressin concentration. As shown in Fig. 2b, the sensor’s resistance decreased over 14 min in response to the decrease in vasopressin levels. Upon reaching a pre-determined 15% decrease in vasopressin concentration, corresponding to 0.25% resistance decrease ratio, the drug delivery is activated autonomously by the application of 35 V between the top and bottom electrodes which results in the release of 16 µL of highly concentrated (200 µM) vasopressin. The localized vasopressin release was detected successfully by the sensor as can be seen in Fig. 2b. This temporary increase in vasopressin would allow a patient to survive until further medical assistance can be provided.

The data obtained from the sensor is in the form of a voltage at the supplied current of 60 mA. This can be transformed into a resistance change that is related to the vasopressin concentration change in solution via the following experimentally derived equation:
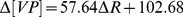
(1)


In the above equation Δ[VP] is in pM and ΔR is a % change in resistance. The relationship between changes in resistance and concentration is further discussed in the next section and is based on experiments presented elsewhere [20].

### Biosensing Ability of Integrated Device

**Figure 3:**
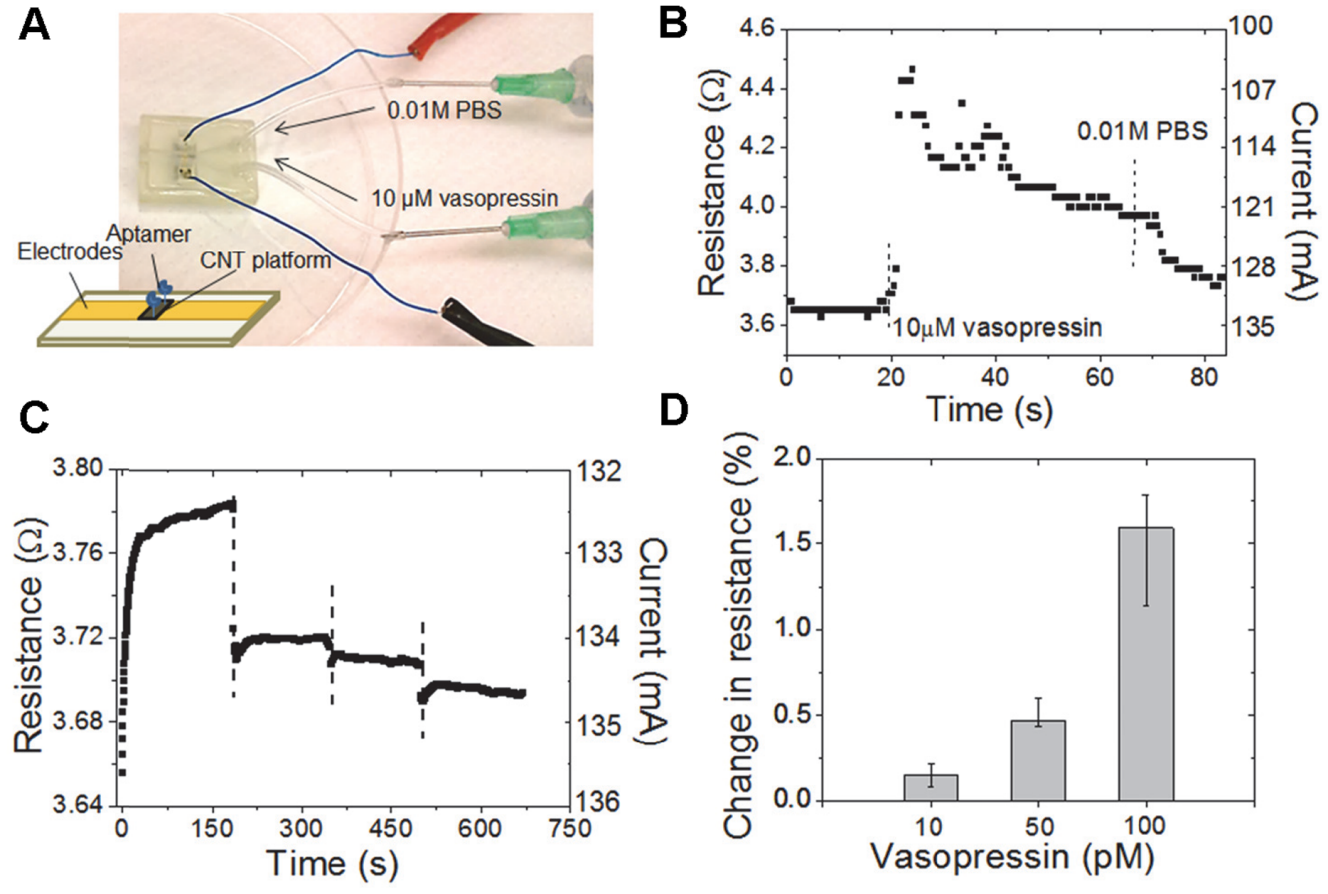
Setup and experiments for sensor characterization. A) Schematic of the nanowire biosensor and picture of the flow through system B) demonstration of sensor reversibility C) resistance, and associated current, change due to continuous decrease in vasopressin concentration in sheep serum. At t = 0 sheep serum with 100 pM vasopressin is introduced, followed by 50 pM, 10 pM and with no additional vasopressin D) Percentage change in resistance from the initial no vasopressin case caused by the flow of sheep serum with different vasopressin concentrations (average of 5 experiments with the error bars representing the highest and lowest reading).

The nanowire biosensor we have developed and integrated in the hemoAID allows for the detection of pM changes in vasopressin concentration. Immobilized aptamer molecules on the nanowire biosensor act as electrical bioreceptors for the surrounding vasopressin. Reversible vasopressin binding leads to changes in conductivity through the CNT detection zone. The sensor is based on a device developed previously with a limit of detection of 43 pM in standard solution [20]. In this section, we demonstrate the sensing ability of the hemoAID for gradual changes in vasopressin levels by performing several characterization experiments. First, we show the reversibility of our sensor’s detection mechanism by monitoring the sensor response to alternating vasopressin concentrations in a single experiment. The biosensor was placed into a custom packaging with micro-channels as shown in Fig. 3a and 0.01 M Phosphate Buffer Saline (PBS) solution was introduced through one inlet until the output current was stabilized. The flow of 0.01 M PBS was stopped after 20 s and 10 µM vasopressin was allowed to flow through the other inlet, which was again replaced by a PBS injection after another 45 s. As shown in Fig. 3b, the initial introduction of a high-concentration 10 µM vasopressin solution results in a current decrease, and resistance increase, through the sensor almost instantaneously which is a consequence of depletion of charge carriers on the CNTs due to vasopressin to aptamer binding. The recovery of current upon 0.01 M PBS injection at t = 50 s is due to the release of vasopressin molecules from the immobilized aptamer which demonstrates the reversible nature of the binding events. The slower rate of current change during the dilution is due to the low dissociation constant of aptamer-vasopressin complex, confirming the high affinity of aptamer molecules to vasopressin [26]. This experiment demonstrates the biosensor’s rapid reversibility even when a high vasopressin concentration causes large changes in resistance, uncommon under normal physiological conditions.

In addition, we show that the sensor can be used to monitor physiologically relevant changes in serum vasopressin concentration. In Fig. 3c, the biosensor is subjected to continuous variations in vasopressin by flowing sheep serum (Valley Biomedical Inc.) with 100 pM, 50 pM, 10 pM and 0 pM of added vasopressin in the respective order for a period of 150 s at each concentration. With each dilution, the current through the sensor increases as expected due to the release of vasopressin from the immobilized aptamers. In Fig. 3d we show the percentage change in current from the initial no vasopressin case due to the addition of 10 pM, 50 pM and 100 pM vasopressin. The bar graph represents the average of 5 experiments that were performed by continuously varying the concentration of vasopressin as shown in Fig. 3c, with the error bar representing the range between the highest and lowest reading. These experiments demonstrate that the sensor can detect physiologically relevant changes in vasopressin in serum.

The high current used to increase the sensitivity of the sensor in the previous section can be decreased by changing the design of the sensor to increase its resistance either by decreasing the thickness of the nanowire sensing area on which APTMS is immobilized or by integrating several such nanowires in series. With these improvements, the sensor can be used for detection of vasopressin in interstitial fluid where changes in vasopressin are gradual and not affected by pressure changes in the bloodstream.

### Stabilizing Vasopressin Levels through Drug Delivery

**Figure 4:**
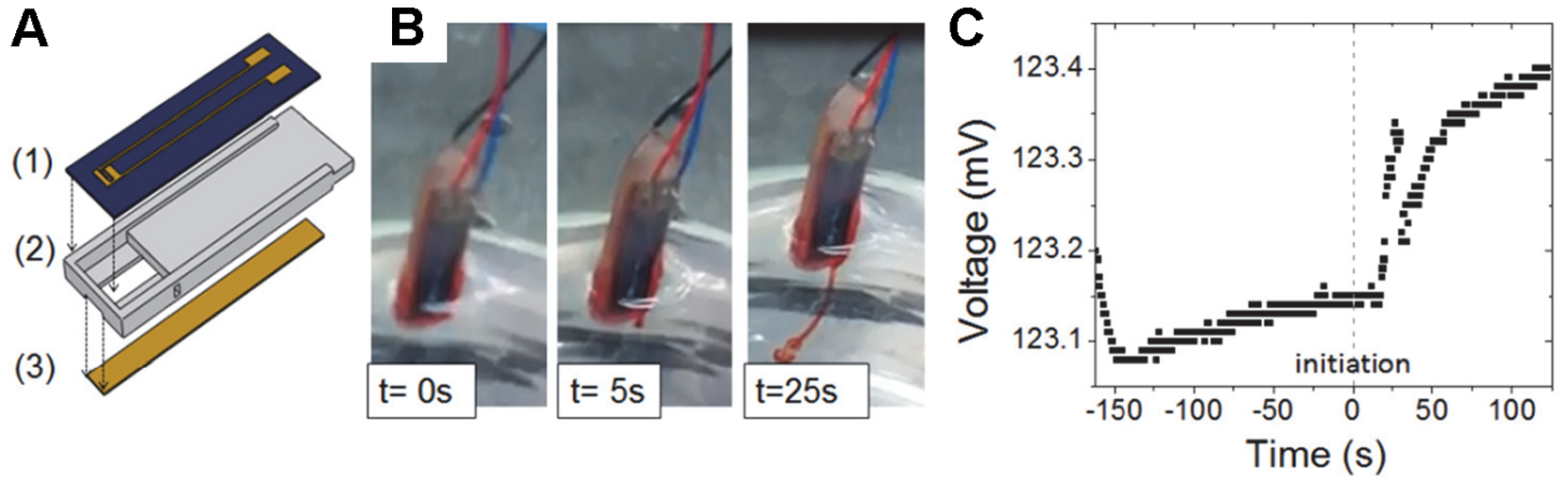
Drug delivery device activation. A) Assembly of the drug delivery unit B) vasopressin ejection over 25 s of applied voltage C) increase in biosensor voltage due to drug delivery. The vertical dotted line indicates when the potential was applied (at t = 0 s) and the a sustained spike in voltage occurs after 25 s.

In this section we demonstrate the integrated device’s mechanism to stabilize vasopressin levels through the in-parallel operation of the drug delivery system (Fig. 4a) with the biosensor. Time-controlled ejection of vasopressin based on electrochemical principles as shown in Fig. 4b has been demonstrated in previous publications [13], [27]. To release vasopressin, electrical potential is applied between the top and bottom electrodes which results in two distinct reactions. The first is the electrolytic reaction in the reservoir that causes gas bubble formation and pressure build-up as a result. The second is the reaction between the chlorine ions in the buffer solution and the gold capping membrane to form a water soluble complex that gradually dissolves until it ruptures with increasing pressure of the reservoir. In the experiment presented in Fig. 4c, the sensor and the capping membrane of the drug delivery system were aligned 50 mm apart using a custom holder and immersed in a 0.01 M PBS solution. After approximately 162 s, the voltage output has stabilized (at t = 0 s on the graph), and the drug delivery was initiated by the application of a 35 V potential between the top and bottom electrodes of the drug delivery system. The electrochemical ejection procedure has been imaged to suggests the onset of gold membrane rupture 5 s after the potential application [27] and a sustained spike in voltage is first observed after 25 s. The small magnitude of this signal (0.25 mV) and the slow response of the sensor are due to large distance between the sensor and the drug delivery device. In the integrated hemoAID device, and the experiment in Fig. 2b the sensor is placed 5 mm in front of the sensor and the release of 16 µL vasopressin solution at 200 µM causes a much more rapid and larger voltage increase of 3.7%. This experiment demonstrates the ability of the sensor to monitor sudden changes in vasopressin concentration due to drug delivery by measuring the increase in voltage even when the two components are not in direct proximity.

### Powering of Integrated Device

**Figure 5:**
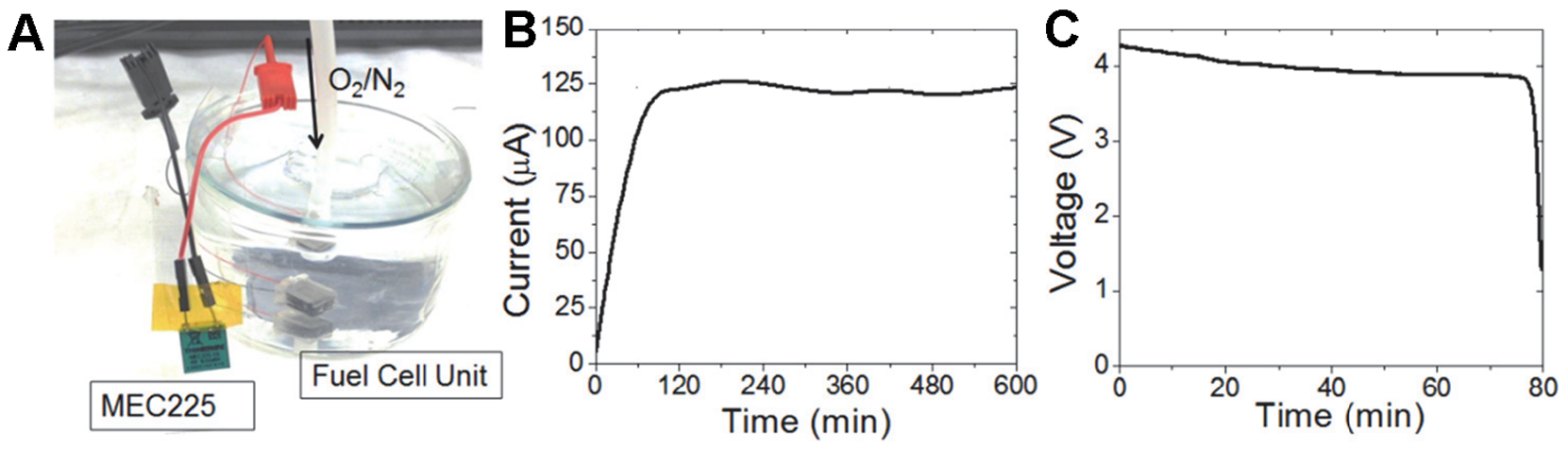
Power unit characterization. A) Power unit consisting of a 1 cm^3^ fuel cell unit in 5 mmol glucose solution at 8% O_2_ saturation and an external rechargeable battery B) chronoamperometric response of the fuel cell unit at 0.1 V in 0.01 M PBS solution and 5 mmol/L glucose solution over a period of 10 h C) discharge profile for the rechargeable battery at an operating current of 100 µA.

Instead of using the fuel cell unit directly to power the device, a small rechargeable battery shown in Fig. 5a is used as an intermediate in order to decrease power supply fluctuations. In addition this also allows excess energy to be stored and used for the one-time operation of the drug delivery device.

The power consumption for the integrated device with the sensor operated continuously is around 60 mA at 3 V. This is well above what can be supplied by a 1 cm^3^ fuel cell unit (20 µA at 0.15 V) however most of the power is required by the biosensor since the change in resistance across the detection area is directly proportional to the current applied. Although decreasing the current supply is in theory possible, this results in lower detection sensitivity. However, the microcontroller only requires 60 to 70 µA at 3 V to operate and we can operate the sensor at discrete time intervals, thus greatly reducing the power consumption of the system and making it possible to operate the system using a 1 cm^3^ fuel cell unit. In Fig. 5b we show the chronoamperometric response of the fuel cell unit (composed of 12 stacked single layers) at 0.1 V in 0.01 M PBS solution (pH 7.4) and physiological levels of glucose and oxygen (5 mM glucose, 8% oxygen saturation) over a period of 10 h. In Fig. 5c we show the discharge profile of the Thinergy MEC225 with an operating current of 100 µA. By continuously charging the Thinergy battery the system can operate continuously over a long period of time and avoid the power fluctuations typical of glucose fuel cell units (±4% as can be seen in Fig. 5b).

## Conclusion and Future Perspectives

In this paper, we have introduced the hemoAID, a device that can be used to detect a gradual drop in vasopressin concentration, corresponding to the onset of late-phase hemorrhagic shock, and deliver a highly concentrated dose of vasopressin to help stabilize the situation. We have demonstrated its operation in a controlled fluidic environment in which a 15% drop in vasopressin concentration was sensed and automatically triggered the activation of the drug delivery system. We discussed the sensor’s mechanism and ability to operate in a complex medium such as sheep serum thus opening the door to the development of an implantable device for late phase hemorrhagic shock prevention.

Miniaturization and i*n-vivo* testing are the next steps in the development of an implantable hemoAID. The operation of the individual components of this device, or similar technologies, have been demonstrated elsewhere *in-vivo* or in serum. The vasopressin drug delivery system presented here is based on a device, by Chung *et al.*, that allows controlling the rate of drug ejection and has been later shown to operate *in-vivo* in *Manduca sexta* moths [28]. In that work the drug delivery devices are sealed using biocompatible wax and implanted in the moths for over 2 days demonstrating stability of vasopressin during storage and upon electrochemical ejection. Other groups have developed similar micro-electro-mechanical-systems (MEMS) for rapid delivery of vasopressin and have demonstrated biocompatibility and long term vasopressin storage without degradation [29]. Some preliminary *in-vivo* studies in rabbits have been reported by H. L. Ho. Duc where a 20 µL dose of 100 µg/kg vasopressin solution is delivered subcutaneously and an increase in plasma vasopressin levels was observed to reach a stable concentration after about 1 h [30]. These experiments demonstrate that such drug delivery technology is suitable for implantation and that the amount of vasopressin required is not an obstacle. The hemoAID reservoir contains approximately 16 µL of concentrated vasopressin solution (200 µM) or approximately 1.4 IU. When the device is loaded with a saturated solution of Arg8-vasopressin (400 IU/mg with 20 mg/ml solubility [31]) it is able to deliver a maximum of 128 IU. This falls within the range of vasopressin levels required to temporarily maintain a late-HS patient alive as discussed previously. Multiple drug delivery reservoirs can be integrated to deliver vasopressin over a longer period of time without substantially increasing the volume of the device as was done by Chung *et al.* [32].

In our previous work we have demonstrated the *in-vivo* operation of the drug delivery system after a 2 day implantation period in moths [28]. For a longer implantation period, as would be required for late phase sceptic shock prevention, a two reservoir system can be used in order to allow the drug to remain in solid lyophilized form until the device is electrochemically activated [30]. The operation principle of the drug delivery system would remain the same; however one reservoir would contain the lyophilized drug and the other the reconstruction media. The stability of vasopressin following electrochemical ejection from our device was investigated using MALDI-TOF/TOF mass spectroscopy in the appendix of a previous work [13]. Strong vasopressin peaks were observed for the ejected contents after application of a 12 V potential for 5 min indicating that only a small fraction of the vasopressin was oxidized during the ejection process.

In the current form, the device is integrated in a 3 cm^3^ packaging with the fuel cell occupying about 1 cm^3^ and the circuitry being external to the device. The miniaturization of similar circuitry has been demonstrated by Gumus *et al.* for application in *in-vivo* uric acid sensing on domestic chicken [33]. We envision the final version of the hemoAID, with multiple vasopressin drug delivery reservoirs, on-board circuitry and coated in biocompatible wax, to be suitable for sub-cutaneous implantation.
